# Statistics and Analysis of Digital Information on Vascular Plant Specimens and the History of Plant Collecting in Guangzhou, China

**DOI:** 10.3390/plants12183325

**Published:** 2023-09-20

**Authors:** Miaoting Liang, Xinsheng Qin

**Affiliations:** College of Forestry and Landscape Architecture, South China Agricultural University, Guangzhou 510642, China; liangmiaoting@outlook.com

**Keywords:** Guangzhou, digital plant specimen information, vascular plant resources, plant collection history

## Abstract

This paper presents a comprehensive analysis of digitized specimen data and relevant literature to investigate the vascular plant diversity in Guangzhou City, China. Specimen data were collected from various sources, including the China Digital Herbarium (CVH), the National Specimen Resource Sharing Platform (NSII), Global Plants on JSTOR, and the Global Biodiversity Information Facility (GBIF). Following data standardization, the study identified 41,890 vascular plant specimens, encompassing 248 families, 1563 genera, and 4536 species, including subspecies and cultivated plants. Among them, the native plants of Guangzhou city accounted for 60.6% of the species. The temporal analysis identified three distinct peaks in specimen collection: 1916–1920, 1928–1936, and 1950–1964. Collection activities were primarily concentrated between the months of April and November. The distribution of collected specimens exhibited significant variation among different species, with families such as Fabaceae, Poaceae, and Myrtaceae having the highest number of specimens. Similarly, genera such as *Eucalyptus*, *Ficus*, and *Citrus* were well-represented. The most frequently collected species included *Litchi chinensis*, *Eucalyptus robusta*, and *Cycas taiwaniana*. Remarkably, 21 species had specimen counts exceeding 100. Unfortunately, approximately three-quarters of the species had fewer than 10 recorded specimens. Alarmingly, 1220 species were represented by only one specimen. Geographically, the majority of specimens originated from the former suburbs of Guangzhou, Conghua Delta Mountain, and Liuxi River areas, while other regions had limited representation. In terms of specimen collections, the Herbarium of South China Botanical Garden of the Chinese Academy of Sciences (IBSC) recorded the highest number of specimens (13,828 specimens), followed by the Tree Herbarium of South China Agricultural University (CANT; 3869 specimens) and the Herbarium of Sun Yat-sen University (SYS; 3654 specimens). The collection history in Guangzhou spans nearly 300 years and can be broadly divided into two distinct periods. The first period extends from the late 13th century to 1949, primarily encompassing the collection efforts of foreign visitors in Guangzhou, and represents the pioneering phase of plant taxonomy research in China. The second period, from 1949 to the present, is characterized by extensive investigations and collection activities conducted by local scholars, with a specific emphasis on native plant resources. By meticulously organizing and verifying information derived from historical documents and specimens, the paper effectively summarizes the plant collection and research history of Guangzhou, providing detailed profiles of the key collectors. These findings furnish reliable historical reference materials for the study of plant taxonomy and diversity in Guangzhou.

## 1. Introduction

**Figure 1:**
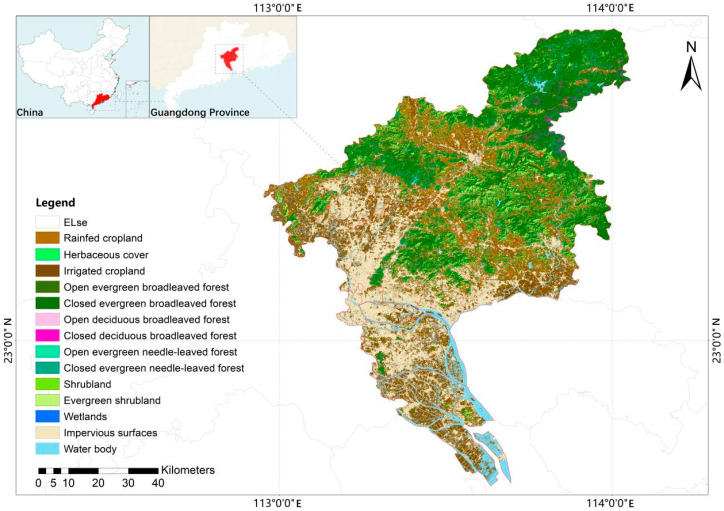
The location and land cover type within and surrounding Guangzhou (Data derived from Liu and Zhang [7]).

Guangzhou is situated in the southern region of China, specifically in the lower reaches of the Pearl River basin, and is located in close proximity to the South China Sea. It is geographically positioned between 112°57′~114°3′ E and 22°26′~23°56′ N, which shares maritime boundaries with Hong Kong and Macao. It is intersected by the Tropic of Cancer and falls within the South Asian tropical monsoon climate zone; both the ocean and the mainland have a significant impact on the climate of Guangzhou. The exceptional natural conditions and intricate geological landforms of Guangzhou have fostered the development of a wide array of plant species and diverse natural vegetation. The region boasts a remarkable forest cover of 42.14% [1]. The predominant zonal vegetation found in Guangzhou is the South subtropical monsoon evergreen broad-leaved forest (Figure 1). These forests are renowned for their lush foliage and abundant biodiversity. Guangzhou also stands out as one of the areas in China with the most abundant fruit tree resources, further exemplifying its ecological richness and agricultural significance [2]. Moreover, Guangzhou holds the distinction of being among the inaugural group of renowned national historical and cultural cities in China. It also stands as a prominent point of origin along the historic Maritime Silk Road, earning it the reputation as a “commercial capital with a legacy spanning millennia”. Throughout antiquity, Guangzhou has served as a hub for the convergence and fusion of Chinese and foreign cultures, establishing itself as the birthplace of Guangfu culture.

Specimens serve as crucial records and custodians of plant collection history, offering direct and objective insights into vegetation status, collection activities, distribution, phenological expression at exact times, and other pertinent information, thereby holding significant importance for scientific research. In the present era, the advancement of computer and internet technologies, as well as the widespread application of information technology in the life sciences, has propelled the process of specimen library informatization. The digitization and informatization of plant specimens have ushered in a new era, ensuring the permanent preservation of essential information pertaining to plant morphology and distribution. This has greatly enhanced information sharing, facilitating the provision of phenology, morphology, distribution, and historical change data to scientific researchers and individuals from diverse backgrounds. These data play a crucial role in understanding the impacts of climate change on plant species [3,4], assessing the spread and ecological influences of invasive species [5,6], and formulating effective conservation strategies. Overall, specimen data serve as a critical tool for advancing scientific knowledge and addressing pressing environmental challenges.

Guangzhou, as a significant trading port, holds the distinction of being the pioneering region where plant collection and research were initiated in China. With a rich history spanning nearly 300 years, Guangzhou’s contributions to plant collection and research have played an exceptionally pivotal role in the field of plant taxonomy, both domestically and globally. The historical significance of plant collection and research in this area remains highly influential, shaping the trajectory of scientific investigations in the realm of plant taxonomy within China and across the world.

This study aims to comprehensively examine the digitalization status of vascular plant specimens in Guangzhou and assess the collection status of plant specimens in the region. The research methodology involves reviewing and referencing relevant literature, consulting university and herbaria archives, and utilizing digitized specimen data available in prominent network databases. By collating and statistically analyzing the specimen information, as well as incorporating data gathered from literature sources, the study presents a comprehensive overview of the research process of plant collections in Guangzhou. Additionally, supplementary details are provided to ensure a comprehensive understanding of the current state of digitalization of vascular plant specimens and the collection status of the area. The findings of this study serve as a valuable scientific foundation for effectively organizing future specimen collection efforts and prioritizing digitization initiatives. Furthermore, the results offer reliable historical reference materials that contribute to the study of plant taxonomy and diversity in Guangzhou.

## 2. Materials and Methods

### 2.1. Data Source

The digital specimen information mainly comes from the Chinese Virtual Herbarium (CVH, http://www.cvh.ac.cn/; last date of access 13 June 2023), the National Specimen Information Infrastructure (NSII, http://www.nsii.org.cn/2017/query.php?kingdom=plant; last date of access 9 June 2023), Global Plants on JSTOR (https://plants.jstor.org/; last date of access 25 June 2023), and the Global Biodiversity Information Facility (GBIF, https://www.gbif.org/; last date of access 1 July 2023). The literature data mainly come from Web of Science (WOS, https://www.webofscience.com/; last date of access 15 July 2023), CNKI (http://www.cnki.net/; last date of access 20 July 2023), the Biodiversity Heritage Library (BHL, https://www.biodiversitylibrary.org/; last date of access 18 July 2023) and Tropicos (https://www.tropicos.org/; last date of access 18 July 2023). A total of 109,288 specimen records collected in Guangzhou between the years 1751 and 2020 have been obtained (Table 1).

### 2.2. Specimen Data Correction

The original specimen data underwent organization using Excel 2021, whereby duplicate records and specimens collected outside the administrative area of Guangzhou were eliminated. Subsequently, the reorganized data will be submitted to the iPlant (http://www.iplant.cn/pnc; last date of access 10 July 2023) and the Taxonomic Name Resolution Service (TNRS, https://tnrs.biendata.org/; last date of access 10 July 2023) for the purpose of correcting and updating specimen scientific names. Finally, the data will be summarized, with angiosperms classified according to the APG IV classification system, gymnosperms classified according to the Gymnosperms classification system, and ferns classified according to the Taxonomic system of stone pines and ferns.

### 2.3. Type Specimen Examination

Utilizing the resources provided by the International Plant Names Index (IPNI, https://www.ipni.org/; last date of access 13 July 2023) and Tropicos (2023), the collection of type specimens from Guangzhou was facilitated. Each type specimen collected from Guangzhou was individually examined and studied, enabling a meticulous analysis of the type species specific to the region.

### 2.4. Standardization of Specimen Data

The standardization of data organization holds tremendous significance within the academic realm. Digitization and organization of specimens occur across various specimen museums, often resulting in disparate data representations and the potential for errors in data entry. Common issues encompass Latin name misspellings, errors in specimen identification, and inconsistencies between webpage data and the original specimen data. Even when recorded by the same individual, discrepancies may arise. For example, the 1974 Guangdong Timber Survey was documented in various formats, such as those of Guangdong Timber, the Guangdong Timber Survey Team, and the Guangdong Timber Survey Team. Complex fonts and illegible handwriting have led to incorrect specimen entries. Notable examples include K.K. Tsoong, misspelled as K.K. Toong, and C.O. Levine, mistakenly listed as Coo. Levine. Furthermore, each database employs distinct input formats. This article undertook the standardization and organization of specimen data collected from the Guangzhou area between 1751 and 2020, utilizing existing data and eliminating duplicates. The processed data facilitated analyses of the collection status of specimens obtained from Guangzhou city, the temporal distribution of plant specimens, and the collection trends among different taxonomic groups. Additionally, the study explored the functional utilization of plants and examined the principal plant collection activities during various periods.

## 3. Results

### 3.1. Temporal Collection Patterns of Specimens in Guangzhou: Yearly and Monthly Variations

**Figure 2:**
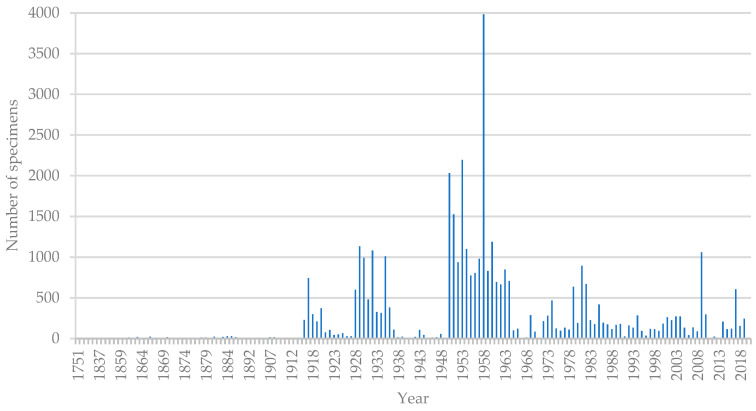
Variation in Yearly Specimen Collection Count in Guangzhou.

Following the standardization of collection dates, a comprehensive dataset comprising 35,149 specimen records with detailed collection dates was obtained. Additionally, 4509 specimens included collection year and month information, 680 specimens solely indicated the year, while 1651 specimens did not have recorded dates. Analysis of the collection years demonstrated distinct patterns of periodicity. Specifically, two minor peaks were observed during 1916–1920 and 1928–1936, while a major peak occurred between 1950 and 1964 (Figure 2). The remaining years exhibited scattered collection patterns without significant periodic trends. Upon consulting relevant literature, significant historical events and large-scale specimen collection activities were identified during the three periods of extensive collection. For instance, in 1916, the establishment of the Agricultural Department at Lingnan School, under the directorship of George W. Groff (1884–1954), facilitated the collection of plant specimens [8]. In 1928, Chen Huanyong was appointed as a professor at the School of Science, National Sun Yat-sen University, and established the “Plant Research Laboratory”. Subsequently, in 1929, the Agricultural and Forestry Plant Research Institute of Agricultural College, National Sun Yat-sen University, established the Plant Herbarium (now known as IBSC) under the directorship of Chen Huanyong, implementing a four-year collection plan [9]. Furthermore, in 1950, the first national conference on plant classification was held in Beijing to discuss the nationwide collection of plant specimens in preparation for the compilation of the Chinese flora. The largest scale of collection occurred in 1958, coinciding with the Sino-German joint investigation conducted during that period [8].

**Figure 3:**
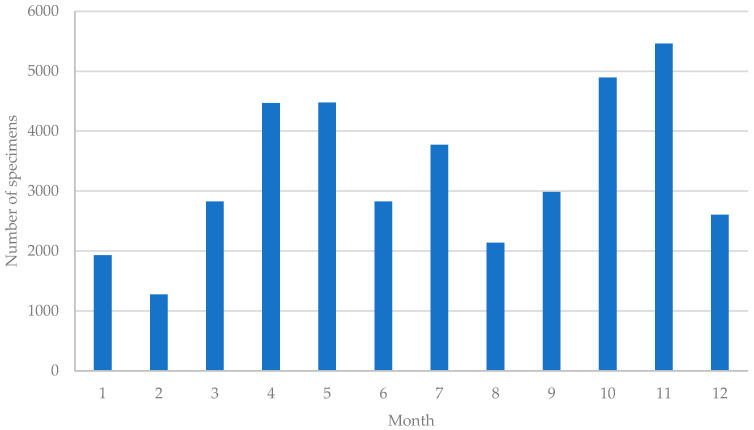
Variation in Monthly Specimen Collection Count in Guangzhou.

The collection of specimens in Guangzhou exhibits a predominant trend from March to November, with the highest collection rates observed from April to May and October to November. Conversely, there are fewer records during January and February. Excluding these two months, the number of specimens collected in other months exceeds 2000 (Figure 3). This pattern is closely intertwined with plant phenology, as specimens often require crucial identification features such as flowers or fruits. The flowering and fruiting stages of plants primarily occur in spring, summer, and autumn, making this period the peak season for specimen collection. In contrast, the relatively cold climate in Guangzhou during January and February results in fewer specimens with distinct characteristics, accompanied by a decrease in plant investigation activities. In general, specimen collection in Guangzhou remains active throughout the year.

### 3.2. Taxonomic Diversity of Specimen Collection in Guangzhou

**Figure 4:**
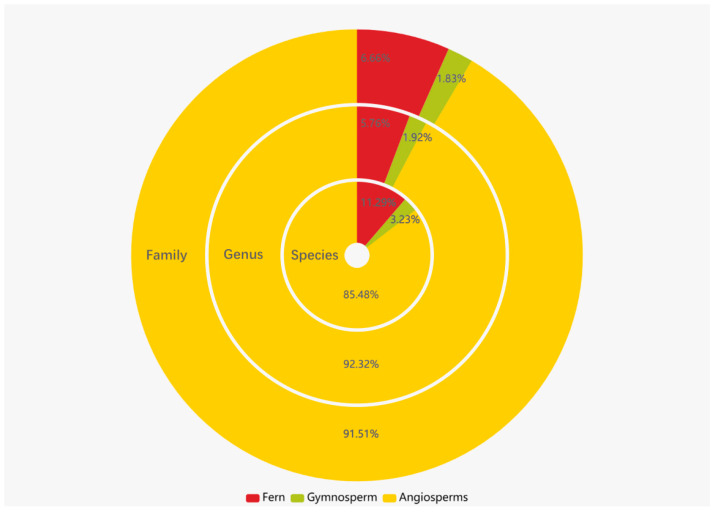
Distribution of vascular plant quantity of Guangzhou.

**Figure 5:**
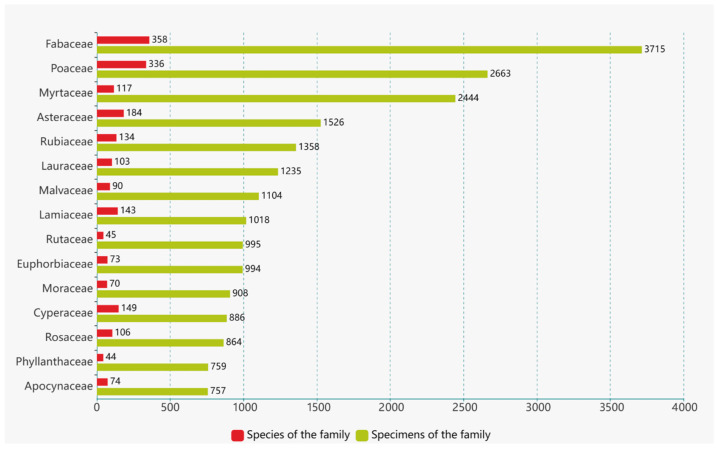
Major Families in the Digital Plant Specimens of Guangzhou.

**Figure 6:**
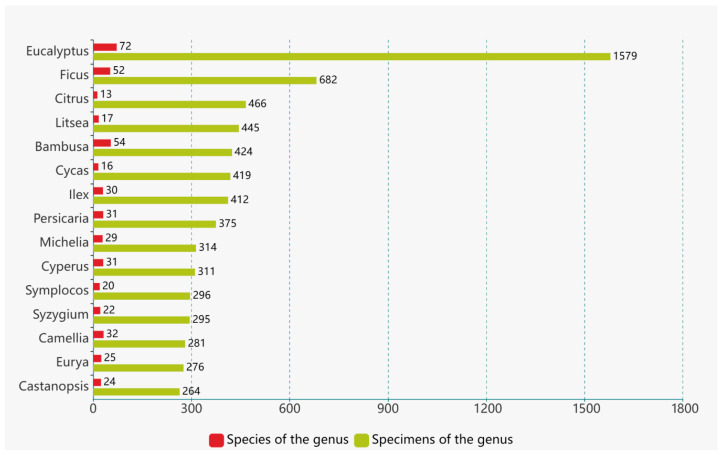
Major Genera in the Digital Plant Specimens of Guangzhou.

**Figure 7:**
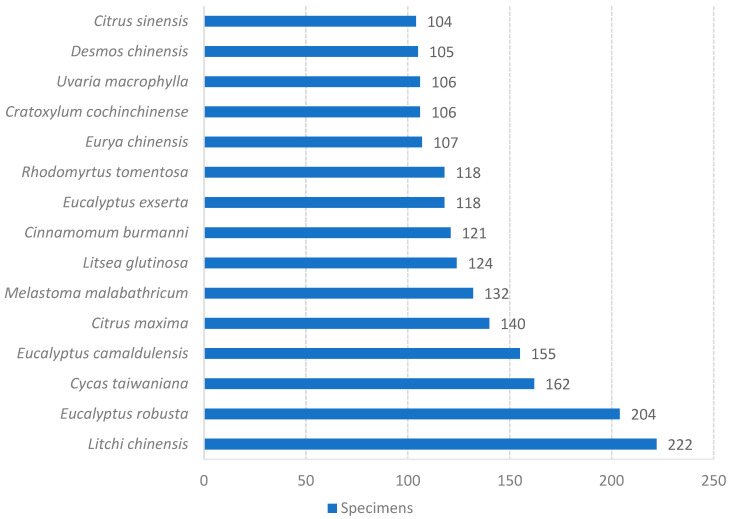
Species with more than 100 Specimens of Guangzhou.

After conducting a thorough name check, a total of 41,890 records of vascular plant specimens were obtained. These records were identified at the species level, encompassing subspecies and cultivated plants. The dataset comprises specimen information for 4536 plant species belonging to 1563 genera and 248 families. Among the specimens, pteridophytes are represented by 28 families, 90 genera, 302 species, and 1761 records. Gymnosperms include 8 families, 30 genera, 83 species, and 1286 records. Angiosperms exhibit the highest diversity, with 212 families, 1443 genera, 4151 species, and 38,843 records (Figure 4). The families with the highest number of specimens include the Fabaceae, Poaceae, Myrtaceae, Asteraceae, and Rubiaceae (Figure 5). Similarly, the genera with notable collection records are *Eucalyptus*, *Ficus*, *Citrus*, *Litsea*, and *Bambusa* (Figure 6). Moreover, the species with the most collection records are *Litchi chinensis*, *Eucalyptus robusta*, *Cycas taiwaniana*, *Eucalyptus camaldulensis*, *Citrus maxima*, and others (see Figure 7).

Among the Pteridophyte specimens, the families Pteridaceae (368 specimens), Athyriaceae (170 specimens), Thelypteridaceae (155 specimens), Polypodiaceae (142 specimens), Dryopteridaceae (118 specimens), and others stand out as having the highest number of standardized specimens. Notably, *Adiantum flabellulatum* (66 specimens), *Palhinhaea cernua* (63 specimens), *Cyclosorus parasiticus* (58 specimens), *Deparia petersenii* (57 specimens), and *Pteris vittata* (55 specimens) represent the species with the largest number of specimens in the collection.

Among the gymnosperm specimens, the greatest representation of collections are from the families Cupressaceae (420 specimens), Cycadaceae (419 specimens), Pinaceae (155 specimens), and Podocarpaceae (118 specimens). Notably, *Cycas taiwaniana* (162 specimens), *Cycas szechuanensis* (88 specimens), *Glyptostrobus pensilis* (83 specimens), *Pinus massoniana* (65 specimens), and *Cunninghamia lanceolata* (62 specimens) are the species with the highest number of specimens.

Among the angiosperm specimens, the families Fabaceae (3175 specimens), Poaceae (2663 specimens), Myrtaceae (2444 specimens), and Asteraceae (1526 specimens) are the most abundant in terms of representation. Notably, the genera *Eucalyptus* (1579 specimens), *Ficus* (682 specimens), and *Citrus* (466 specimens) have the highest number of collections at the genus level. Furthermore, species such as *Litchi chinensis* (222 specimens), *Eucalyptus robusta* (204 specimens), *Eucalyptus camaldulensis* (155 specimens), *Citrus maxima* (140 specimens), *Melastoma malabathricum* (132 specimens), *Litsea glutinosa* (124 specimens), and *Cinnamomum burmanni* (121 specimens) exhibit the most abundant representation at the species level.

In the entire collection of specimens, there are 53 families with fewer than 10 specimens and 9 families with only 1 specimen. Similarly, there are 791 genera with fewer than 10 specimens and 217 genera with only a single collection. Nearly three-quarters of the species have fewer than 10 specimens, with 1220 species represented by only one specimen, accounting for 26.89% of the total species, indicating a low representation. Taxonomic groups or species with a higher number of specimens are often associated with a wider distribution range, making them more likely to attract the attention of collectors.

### 3.3. Analysis of the Diverse Utilization of Native Plants in Guangzhou Based on Specimens

Native plants are defined as plant species that naturally occur in specific regions without human intervention [10]. Based on the geographic distribution of vascular plants, they have been categorized into two distinct groups: native plants and exotic/cultivated plants. In Guangzhou, the database contains 2732 species of native plants, which accounts for 60.2% of the total plant species recorded, with a corresponding collection of 28,759 specimens. Conversely, there are 1804 species of non-native plants, accompanied by 13,131 specimens, representing approximately one-third of the total specimen count (Table 2).

Plant resources encompass the flora that is accessible, potentially exploitable by humans, and also that which can be conserved and stewarded within the constraints of socio-economic and technological circumstances. As the cultural hub of Lingnan, Guangzhou possesses a reservoir of traditional knowledge within the local populace concerning the management, conservation, and utilization of plant diversity. Native plants in Guangzhou have been categorized into eight distinct classifications based on their utilization methods and purposes: edible (starchy), medicinal, toxic, fodder (grazing grass/honey source), fiber, timber, ornamental landscaping, and oil (essential oil/resin/tannin). Empirical research findings exemplify the diversity of functional anthropocentric uses exhibited by indigenous plants in Guangzhou (Table 3). Examples encompass 269 species of edible (starchy) plants, 1395 species of medicinal plants, 67 species of toxic plants, 148 species of taxa used for fodder (grazing grass/honey sources), 109 species of fiber plants, 277 species of timber plants, 647 species of ornamental landscaping plants, and 193 species of oil/fat (or containing essential oil/resin/tannin) plants. Numerous plants in the region possess both nutritional and medicinal value, with 204 species concurrently exhibiting these dual attributes, presenting substantial potential for their horticultural or agricultural development and utilization.

### 3.4. Specimen Collection Sites in Guangzhou

**Figure 8:**
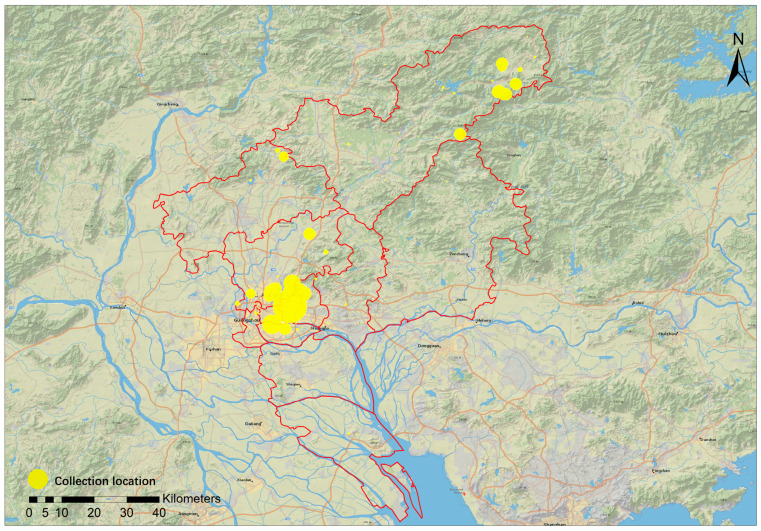
Distribution of the primary specimen collection sites in Guangzhou.

Via verification and organization, a total of 18,916 specimen records containing detailed collection site information were obtained. Visualization of the collection site information reveals that the majority of the specimens were collected from the old outskirts of Guangzhou and places such as Sanjiao Mountain in the Liuxi River of Conghua district (Figure 8). Due to the process of rapid urbanization, previously, suburban areas have been transformed into urban districts. Many of the historically suburban collection sites mentioned in the specimen records, such as Wushan, Shipai, Dongshan, and Kangle Village, now lie within a maze of high-rise buildings. Currently, there are more collection records from sites where vegetation is relatively well-preserved, such as Baiyun Mountain, South China Botanical Garden, and Longyandong Forest Park. Notably, a significant number of specimens were collected near roadsides. The distribution of collection sites provides valuable primary data for understanding the vegetation status and its history in the region and offers a reference for subsequent studies.

### 3.5. Major Herbaria in Which Relevant Collections Are Represented

Based on the currently recorded specimen data, the Herbarium of South China Botanical Garden, Chinese Academy of Sciences (IBSC), holds the foremost position with a collection of 13,829 plant specimens from Guangzhou city. The Plant Herbarium of South China Agricultural University (CANT) curates 3869 collections, and the Herbarium of Sun Yat-sen University (SYS) has 3654 specimens from Guangzhou. The British Museum of Natural History (BM), Muséum National d’Histoire Naturelle in France (NHMUK), Arnold Arboretum (A), New York Botanical Garden Herbarium (NY), Royal Botanic Garden Edinburgh Herbarium (E), and the Missouri Botanical Garden Herbarium (MO) also hold a substantial number of specimens collected from Guangzhou city (Table 4). The significance of these institutions’ collections is closely intertwined with the historical establishment of their herbaria.

### 3.6. Guangzhou Municipal Type Specimen Examination

Based on the compiled specimen data and literature information, statistical analysis was conducted on the type specimens of vascular plants collected from Guangzhou, resulting in a catalog of type specimens of vascular plants from Guangzhou. A total of 509 type specimen records were compiled, of which only 185 specimens clearly indicate the type categories, while the remaining specimens either state “Type” or do not specify the categories (Table 5). A total of 332 species, belonging to 95 families and 231 genera, are represented by type specimens collected in Guangzhou. Families to which the type specimens belong include the Poaceae (32 species, 42 specimens), which has the highest representation, followed by Lauraceae (25 species, 42 specimens) and Euphorbiaceae (21 species, 24 specimens). The Phyllanthaceae also had a relatively large number of type specimens, with 20 species and 26 collections. In terms of the number of type specimens held by herbaria, the British Museum of Natural History (BM) ranked first with 115 specimens, followed by the Herbarium of South China Botanical Garden, Chinese Academy of Sciences (IBSC), which housed 66 specimens. More than 2/5 of the type specimens were stored in foreign herbaria or museums. Regarding the collection years and collectors, the highest number of type specimens were collected by Yongquan Li, who collected 29 species between 2005 and 2007. F.A. McClure collected 25 species in the 1930s, followed by W.T. Tsang, with 21 species in 1932 and 1935, and Yourun Lin, who collected 21 species in 1960–1961. Additionally, Millett, Charles, H.F. Hance, and C.O. Levine also contributed a significant number of collections. In terms of the original publications where the type specimens were described, the primary journals and monographs include the Journal of Botany, British and Foreign (40 species), Philippine Journal of Science (27 species), The Botany of Captain Beechey’s Voyage (22 species), Species Plantarum (21 species), and Acta Phytotaxonomica Sinica (18 species). These types of specimens and their data provide fundamental information for biodiversity research in Guangzhou city.

## 4. Brief History of Plant Collection in Guangzhou City and Its Surroundings

As the cultural center of Lingnan and the sole port for foreign trade during the Qing Dynasty, Guangzhou holds significant importance in the study of Chinese plant taxonomy. Given that plant taxonomy is a product closely aligned with human societal development, this article aims to organize and provide a concise supplement to the botanical collection history of Guangzhou in chronological order by analyzing specimens and reviewing relevant literature while considering the developmental stages of Chinese society and beginning with the year 1949 the date of the establishment of the People’s Republic of China. The botanical collection history of Guangzhou city can be divided into two distinct periods. The first period extends from the late 13th century up until 1949, primarily characterized by the collection activities of foreigners in Guangzhou and the pioneering phase of Chinese plant taxonomy research. The second period encompasses the time from 1949 to the present, primarily focusing on the extensive investigations and collection activities carried out by Chinese scholars independently in relation to indigenous plant resources [11].

### 4.1. History of Plant Collection in Guangzhou before 1949

#### 4.1.1. The Contribution of Westerners to the Plant Collection History of Guangzhou

**Figure 9:**
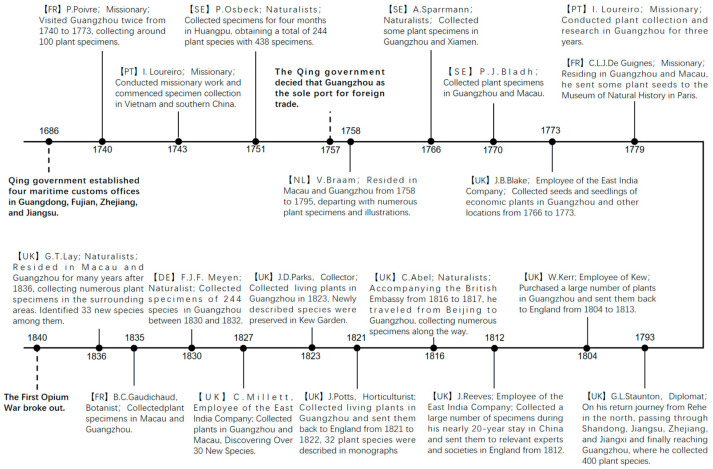
Collection History of Westerners in Guangzhou before the First Opium War. Boldface characters represent historical events. DE = Germany, FR = France, NL = Netherlands, PT = Portugal, SE = Sweden, UK = United Kingdom.

Before the Opium Wars (1839 and 1856), the scope of activities for Westerners in China was very limited, especially after 1757, when Guangzhou became the sole port for foreign trade. Consequently, the plant collection areas for plant collectors were confined to the southeastern coastal region, primarily centered around Guangzhou. Before the mid-18th century period, individuals such as missionaries (including those associated with the court) and diplomatic envoys who engaged in plant collection endeavors in Guangzhou were not specialized botanists. Nonetheless, they had received exceptional education in the humanities and languages, enabling them to engage in exchanges with the imperial court, scholars, and various societal strata in China. Plant collection activities were ancillary to cultural and religious communication, with specialized endeavors in plant collection being relatively scarce. From the latter half of the 18th century onwards, numerous nations began dispatching plant hunters to Guangzhou for the purpose of botanical collection. Most of the plant hunters underwent specialized training in the fields of botany and horticulture and were employed by European scientific establishments, botanical commercial networks, East India companies, and other analogous institutions. Their motivations were driven by both commercial interests and the acquisition of knowledge, with cultural exchange playing a less prominent role. Although most of them engaged only in scattered collection activities in Guangzhou, influential collectors emerged among them, opening the door to understanding Chinese plants for the world. Portuguese missionary J. Loureiro developed an interest in plants during his missionary work in southern China in 1743. In 1779, he spent three years in Guangzhou collecting and studying plants, and upon his return to Portugal in 1782, he published the *Flora Cochinchinensis* [12], which included 539 species of Chinese plants. P. Osbeck, a student of Carl Linnaeus, arrived in Huangpu, Guangdong, on a Swedish East India Company merchant ship in August 1751. He collected plants in the port city for four months, obtaining 244 species with 438 collections. He later gave his collections to Linnaeus, who described 37 species based on Osbeck’s specimens in Species Plantarum. Osbeck himself described 21 new species, including the new genus *Osbeckia* L. and the new species *Clematis chinensis* Osbeck. To further expand commercial opportunities in China, the United Kingdom dispatched diplomatic missions to the country in 1792. Since then, the recorded collection has shown a significant increase in the number of British collectors. Among them, J. Reeves of the British East India Company, who served as a tea inspector in Guangzhou from ca. 1812 to the 1840s, collected a large number of botanical specimens and sent them to relevant experts and societies in Britain during his nearly 20-year stay in China. Additionally, he introduced a large number of garden plants from the outskirts of Guangzhou to Britain, earning great respect from the British scientific community [13]. German naturalist F.J.F. Meyen collected as many as 244 plant specimens (preserved in Berlin) during his voyage on the Prinz ship from 1830 to 1832, including *Clematis meyeniana* Walp., during his time in Guangzhou (Figure 9).

**Figure 10:**
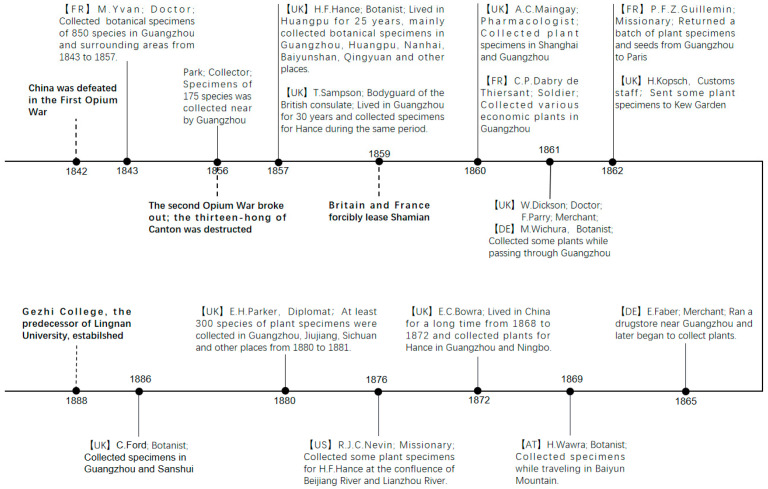
Collection History of Westerners in Guangzhou after the First Opium War. Boldface characters represent historical events. AT = Austria, DE = Germany, FR = France, UK = United Kingdom, US = United States.

After the Opium War, China initiated the process of territorial cession, compensation, and tariff negotiations with foreign powers. This period witnessed China’s transformation into a semi-colonial and semi-feudal society, resulting in the erosion of its independent status. Concurrently, Westerners gradually gained unrestricted mobility within China, facilitating their unrestricted travel throughout mainland China. Capitalizing on this exceptional opportunity, Western individuals were dispatched to China not only to introduce valuable plant resources but also to undertake botanical investigations in accessible cities and their surrounding environments. Among these individuals, H.F. Hance, the British Consul in Huangpu, emerged as a notable figure. Combining his roles as a British diplomat and a plant taxonomist, Hance actively engaged in the extensive collection of plants in southern China, playing a pioneering role in botanical collection endeavors. Joining the Guangzhou Consulate in 1856, he subsequently resided in Huangpu, Guangzhou, for 25 years after his arrival in Xiamen in 1857. Hance succeeded in persuading numerous Westerners residing in China to assist him in procuring plant specimens, resulting in the establishment of a substantial collection network that encompassed individuals such as F. Parry, R.J.C. Nevin, E.H. Parker, T. Sampson, H. Wawra, and others [13]. Between 1843 and 1857, Dr. M. Yvan from the French Embassy in China undertook an extensive collection of plant specimens in Guangzhou and its nearby areas, organizing local individuals to participate in the collection efforts, and a total of 850 species of specimens (excluding ferns) were amassed during this period (Figure 10).

**Figure 11:**
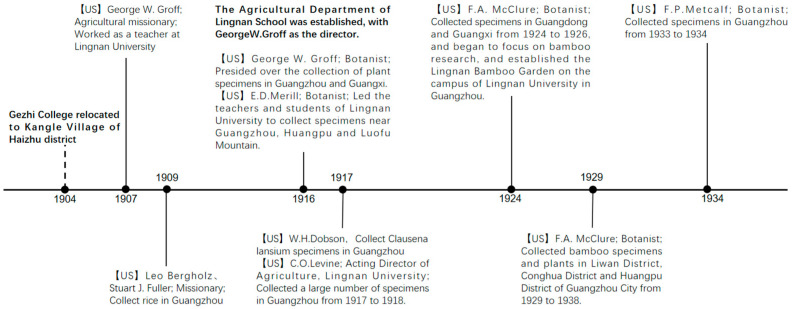
Collection History of Westerners in Guangzhou after 1900. Boldface characters represent historical events. US = United States.

Influenced by botanical collection activities conducted by Britain, France, and other countries, the United States embarked on its own collection endeavors in China. During the early 20th century, a large-scale agricultural missionary movement was carried out by the United States. The American government invited experienced plant hunters and botanists to conduct extensive collection activities in China. Many American scholars carried out their botanical research and plant specimen collection in Guangzhou, with Lingnan University serving as their primary base of operations. E.D. Merrill, a renowned botanist and Dean of the School of Agriculture at the University of California, USA, is among the most distinguished scholars in the field of Chinese plant studies. He arrived in Guangzhou in 1916 and led a group of teachers and students from Lingnan University to collect numerous specimens in the vicinity of Guangzhou, Huangpu, and Luofu Mountain. These collections later formed the foundation of Lingnan University’s specimen room [13]. Another significant scholar is George W. Groff. In 1916, the Agronomy Department of Lingnan School was established, with Groff serving as its director, and oversaw the collection of plant specimens in Guangzhou and Guangxi. An American botanist named F.A. McClure, also known as Mo Guli in Chinese, taught at Lingnan University for many years. Beginning in 1924, he dedicated three consecutive years to collecting bamboo specimens in Guangdong and Guangxi, delving into bamboo research. During this period, McClure visited various districts in Guangzhou city, including Baiyun, Haizhu, Huangpu, and Liwan, and established the Lingnan Bamboo Garden on the campus of Guangzhou Kangle Lingnan University (now known as Sun Yat-sen University). From 1929 to 1938, many specimens of bamboo and other plants were collected by McClure in different districts of Guangzhou (Figure 11).

The endeavors of plant collectors traversing the lands of China and successfully transporting plants to the West via Chinese Customs are intricately linked to the protection and advancement of Western powers in China. This symbiotic relationship between foreign plant collection and the expansion of Western influence in China underscores the unique context of species migration during this period. The impact of foreign collectors’ plant collection efforts in Guangzhou, in particular, has been substantial. Over 30 individuals from eight different countries have amassed and exported a significant number of Chinese plant specimens from Guangzhou, including seeds, underground stems, roots, and live plants. Consequently, a vast collection of China’s earliest plant specimens, including type specimens, now reside abroad, with the majority of identifications being conducted by foreign experts. This inevitably led to the initial dissemination of a considerable amount of botanical literature abroad. Furthermore, the introduction of numerous economic plants from Guangzhou has facilitated their dissemination to various regions across the globe. Firstly, ornamental plants, including flowers, hold a prominent position. Guangzhou, renowned as the “Flower City,” boasts a flourishing flower horticulture industry. Prior to the Opium War, the southeastern outskirts of Guangzhou featured a sizable garden area known as Flower Land, which served as a prominent trading hub for flower seedlings and bonsai. Consequently, among the early Western introductions of garden plants, flowers from Flower Land constituted a significant proportion. Secondly, fruit trees also played a vital role. As early as the first half of the 16th century, the Portuguese brought high-quality sweet oranges from China back to Lisbon for cultivation as fruit trees. Another highly sought-after plant was tea. Swedish botanist A. Sparrmann collected over 20 plant species, including rice, camellia, and oranges, from the coasts of Guangdong and Macao. In 1780, ships of the East India Company transported a small quantity of tea from Guangzhou to India for cultivation, marking the first instance of tea cultivation in India. By 1820, China’s tea exports accounted for three-quarters of its overall exports. In that year, the value of Guangzhou’s export trade to Britain and the United States amounted to 13,616,691 yuan, with tea contributing 8,757,471 yuan [14].

#### 4.1.2. Collection History of Chinese Scholars in Guangzhou

Before the Opium War, numerous foreigners arrived in China to explore and collect plants, while they did not impart modern plant taxonomy to the Chinese. Following the signing of the Treaty of Nanjing in 1842, Western culture began to make its way into China. Nonetheless, throughout the 19th century, the Chinese did not actively engage in the collection and research of modern plant taxonomy. It was not until the early 20th century that significant strides were made in this field. From the early 20th century until 1949, pioneers of plant taxonomy in China embarked on extensive specimen collection efforts and conducted preliminary botanical research.

K.K. Tsoong was the first Chinese scholar to systematically investigate and collect higher plants using scientific methods. His collection of plant specimens from 1918 to 1921 marked the inaugural large-scale collection effort undertaken by Chinese scholars. He selected collection sites that were pivotal for foreign collectors, including Guangzhou, Fuzhou, Xiamen, and others. These collections provided Chinese scholars with firsthand data for comprehending the distribution of vegetation in China, particularly in the southern regions [15]. In 1918, botanist W.T. Swingle employed W.S. Kwok, a graduate of Lingnan University, as an assistant to aid in surveying the resource distribution of wild citrus in the Lingnan area [16]. During this period, W.S. Kwok amassed a significant number of citrus specimens. In 1927, Chen Huanyong joined Sun Yat-sen University as a professor. That year, he conducted specimen collection expeditions to northern Guangdong, Guangzhou, Dinghushan, Hong Kong, Guangxi, Guizhou, and other locations. Through his diligent efforts, the Agricultural College of Sun Yat-sen University established the first professional herbarium in South China in 1928. Subsequently, the Plant Research Office of Sun Yat-sen University (later renamed the Institute of Agricultural and Forestry Plant of Sun Yat-sen University) was established, which served as the precursor to the current South China Botanical Garden of the Chinese Academy of Sciences. With the objective of compiling the Guangdong Flora, a four-year plant collection plan for Guangdong Province was formulated by Chen Huanyong. According to the initial five-year report of the Institute of Agriculture, Forestry, and Plants of National Sun Yat-sen University in 1934, collection teams were dispatched to Hainan Island, Hong Kong, and Guangdong 109 times between 1927 and 1933. Following the establishment of the Plant Research Laboratory in 1928, the collection work was primarily undertaken by Y.Tsiang and C.L.Tso. As the institute expanded, the team size grew, with notable collectors including Y.Tsiang, C.L.Tso, N.K.Chun, C.Wang, S.P.Ko, F.C.How, and others (Table 6). This period marked the transition to large-scale collection efforts, with numerous specimens being gathered in Guangzhou between 1928 and 1936 by these collectors and their teams [17].

### 4.2. History of Plant Collection in Guangzhou after 1949

After the establishment of the People’s Republic of China, the investigation, collection, and research of plant resources in the country entered a highly dynamic phase characterized by unprecedented scale and diverse forms and contents of activities. In 1958, a Sino-German expedition jointly organized by the Chinese Academy of Sciences and the German Democratic Republic conducted specimen collection in Conghua. Subsequently, in 1959, in line with the directives outlined in the report on the comprehensive survey and utilization of wild economic plant resources and the compilation of economic flora by the Chinese Academy of Sciences and the Ministry of Commerce, a nationwide general survey of wild economic plant resources was initiated. The South China Institute of Botany participated in the investigation of Guangdong and Hunan provinces. The primary investigation site in the Guangzhou area was Conghua, and the collector assigned to this region was Shaoqing Chen. In the 1970s, a nationwide campaign was launched to investigate the resources of Chinese herbal medicine. The investigation in Guangdong involved the participation of several dozen personnel from the South China Institute of Botany of the Chinese Academy of Sciences. In Guangzhou, collection activities were conducted by the Chinese Herbal Medicine Group, Guangzhou Medicine Group 1, Guangzhou Medicine Group 2, and other teams. In 2004, the Guangzhou Forestry Bureau officially initiated a comprehensive background survey of terrestrial wildlife resources in Guangzhou, which spanned nearly four years. This survey represented the most extensive and systematic assessment of terrestrial wildlife resources since the establishment of the People’s Republic of China. During this period, over 1500 plant specimens were collected, contributing to a comprehensive understanding of the status of terrestrial wildlife resources in Guangzhou [18]. In 2009, the research project on the status of rural fengshui forests and zonal vegetation restoration in the nature conservation community in Guangzhou was initiated. Led by Zhengchun Xu from the School of Forestry and Landscape Architecture at South China Agricultural University, a comprehensive survey was conducted throughout Guangzhou. A total of 12,200 plant specimens were collected, with voucher specimens deposited in the Herbarium of the South China Botanical Garden of the Chinese Academy of Sciences (IBSC) and the Herbarium of South China Agricultural University (CANT) [19]. In addition to the aforementioned major national or regional survey and collection activities, numerous small-scale collection initiatives have taken place involving researchers such as Huang Zhi, Chen Shaoqing, Deng Liang, Ye Huagu, and others (Table 7) [9]. Furthermore, academic institutions in Guangzhou, including South China Normal University and South China Agricultural University, regularly lead students in collecting samples within the city. The information derived from these collected specimens serves as a foundational basis for biodiversity studies in Guangzhou.

## 5. Discussion

The digitization of specimens serves multiple purposes in botanical research and related fields. It not only provides objective, comprehensive, and accurate data but also mitigates the risk of losing crucial information regarding habitat, ecology, geography, and other vital aspects due to specimen destruction. To ensure the meticulous digitization of specimen information, it is essential to maintain detailed and precise records and exercise caution during label recording, identification, and input of specimen data [20]. Although efforts have been made to standardize existing digital specimen information, this study reveals numerous errors and omissions in the current dataset. Examples of these discrepancies include inconsistencies between website and webpage data and specimen photos, data entry errors, blurry images, and disordered collection team or individual names. There are observed variations in the number of specimens collected across different seasons, particularly during the months of January and February. This discrepancy can be attributed to several factors, including the lower temperatures during this period, making field collection more challenging. Additionally, the occurrence of the Lunar New Year, a significant cultural holiday in the region, may lead to reduced collection efforts as researchers and collectors may have less availability or engagement during this time. Furthermore, there is a lack of uniformity in data entry standards across different data-sharing platforms. Consequently, the full potential of existing digital information remains unrealized. It is important to note that the majority of digital specimen information available in open network databases predominantly consists of data collected many years ago, with limited representation of recent years.

Despite numerous plant collection activities conducted in Guangzhou, a majority of collection sites are concentrated in easily accessible areas such as Longyan Cave, Baiyun Mountain, Sanjiao Mountain, and botanical gardens. Further, the plant habitats sampled are often in close proximity to roadsides or within the confines of these gardens. The existing report shows that Guangzhou has various vegetation types, rich species composition, and complex community structure, with tropical and subtropical families such as Theaceae, Fagaceae, Moraceae, Lauraceae, Euphorbiaceae, and Rubiaceae as the dominant families. However, in the existing digital specimen data, the number of specimens of Theaceae, Fagaceae, Moraceae, and other plant families is much smaller than that of Poaceae, Myrtaceae, and Asteraceae [21]. The frequency of plant collections varies significantly among different species in Guangzhou, with some species having over a hundred recorded specimens. However, for 238 genera and 1222 plant species, there is only one recorded specimen. Several potential reasons may assist in explaining this disparity. Some of the genera, such as *Carex*, *Machilus*, and *Bambusa*, present challenges in terms of identification, which may result in a smaller number of collected and identified specimens for species within these genera. Secondly, there is a possibility of identification errors. Thirdly, some species may have incorrect collection location information [22]. Lastly, it may be because the habitat of some plants is dangerous, or the height under the branches of some plants is too high, which makes it difficult to collect specimens, so the number of specimens in some families is small.

Type specimens play a fundamental role in establishing the scientific names of plants. However, organizing, verifying, and digitizing these specimens still presents certain challenges. Various issues arise during the textual research and organization of type specimens. For instance, early type specimens often lack specific designations, merely indicating “Type” without specifying the particular type to which it refers. Currently, only 332 type specimens collected from Guangzhou can be found in commonly accessible databases. This limited number may be attributed to difficulties in obtaining information on overseas specimens and the incomplete digitization of domestic specimens.

## 6. Suggestions and Recommendations

Via the comprehensive organization and analysis of digital specimen data in Guangzhou, we have identified certain shortcomings in specimen collection practices. These findings provide valuable insights for future collection efforts. It is crucial to maintain detailed and accurate original records during the specimen collection process, as they serve as vital evidence for subsequent research. Therefore, adopting a standardized format for data entry is essential to ensure the precision of specimen data. Furthermore, it is imperative to incorporate additional specimen data from recent years to enhance the comprehensiveness of the digital collection. The advent of digital imaging has facilitated the integration of Guangzhou collections into national and international herbarium databases, thereby enabling seamless collaboration among researchers. The utilization of imaging techniques also allows for the assessment of phenological attributes, such as flowering time and other phenological characteristics. These data are invaluable for studying the impacts of climate change over time, as evidenced by observed shifts in phenological patterns. This emerging research domain has garnered international attention, with notable examples including the California Phenology Project and herbarium-based studies conducted within the California Consortium of Herbaria [23,24,25]. Consequently, it is imperative to expand the collection of plant specimens across various seasons, as this will further enrich the knowledge base and enhance the scope of research in this field. Further, particular attention should be given to species and taxa with limited representation in the digital records. Species with insufficient digital records should undergo re-identification annotation or have their information carefully reviewed. If the re-identification process aligns with the existing digital information, it is necessary to prioritize the collection of additional specimens for such species. Conversely, taxa with an excessive number of specimens should be subject to reduction or cessation of collection efforts. Additionally, locations with few collections of specimens but rich vegetation should be targeted for supplementary re-collection activities. Lastly, it is crucial to emphasize the collection of type specimen data stored abroad. Further in-depth research is warranted to explore and investigate type specimens in greater detail. Overall, these recommendations aim to optimize future collection work, enhance the accuracy and comprehensiveness of the digital specimen database, and promote a more robust understanding of plant resources in Guangzhou.

## 7. Conclusions

Guangzhou boasts abundant plant species resources, indicating significant potential for further development and utilization. Moreover, Guangzhou’s significant trading history adds to its prominence as a leading area for plant collection research in China. It has been reported that the total number of wild vascular plant species in Guangzhou is documented to be 3508, a figure that aligns with the digital specimen data available for the region, excluding cultivated species [21]. Despite the progress made in specimen collection, there are still several shortcomings that require individual attention and resolution by scholars. Addressing them will contribute to improving the overall quality and effectiveness of specimen collection efforts.

## Figures and Tables

**Table 1:** Data source.

Websites	Search Condition	No. of Specimens
CVH, http://www.cvh.ac.cn/ (accessed on 13 June 2023)	Country = China, Province = Guangdong, County = Guangzhou/Conghua/Zengcheng/Huangpu/Panyu/Tianhe/Yuexiu/Huadu/Haizhu/Nansha/Baiyun/Liwan/Canton. Country = China, Province = Guangdong, Location cite = Guangzhou/Conghua/Zengcheng/Huangpu/Panyu/Tianhe/Yuexiu/Huadu/Haizhu/Nansha/Baiyun/Liwan/Canton.	34,059
NSII, http://www.nsii.org.cn/2017/query.php?kingdom=plant (accessed on 9 June 2023)	Location = Guangzhou/Conghua/Zengcheng/Huangpu/Panyu/Tianhe/Yuexiu/Huadu/Haizhu/Nansha/Baiyun/Liwan/Canton.	55,632
JSTOR, https://plants.jstor.org/ (accessed on 25 June 2023)	Country = China, Locality = Guangzhou/Canton;	307
GBIF, https://www.gbif.org/ (accessed on 1 July 2023)	Scientific name = Plantae, Basis of record = Preserved specimen, Country or area = China, Administrative areas = Guangzhou-CHN.6.4.1	19,290

**Table 2:** Collection of Native Plant Specimens in the Guangzhou.

	Ferns	Gymnosperms	Angiosperms	Total
Families	28	5	184	217
Genera	83	10	964	1057
Species	248	15	2469	2732
Specimens	1634	391	26,734	28,759

**Table 3:** Main uses of native plants in Guangzhou.

	Ferns	Gymnosperms	Angiosperms	Total
Edible (Starchy)	10	2	257	269
Medicinal	142	10	1243	1395
Toxic	3	0	64	67
Fodder (Grazing grass/Honey source)	2	0	146	148
Fiber	1	3	105	109
Timber	0	12	265	277
Ornamental landscaping	63	13	571	647
Oil (Essential oil/Resin/Tannin)	0	9	184	193

**Table 4:** A list of herbaria harbors more than 1000 specimens from Guangzhou.

Herbarium Name (International Code)	Specimens
Herbarium of South China Botanical Garden, Chinese Academy of Sciences (IBSC)	13,829
Plant Herbarium of South China Agricultural University (CANT)	3869
Herbarium of Sun Yat-sen University (SYS)	3645
Herbarium, Institute of Botany, Academia Sinica (PE)	3271
Herbarium of College of Life Sciences, South China Normal University (SN)	2471
Herbarium of Guangxi Institute of Botany (IBK)	1961
Herbarium of Kunming Institute of Botany, Chinese Academy of Sciences (KUN)	1806

**Table 5:** Categories of Type specimens in Guangzhou.

Categories	Number
/	152
Type	169
Isotype	95
Holotype	34
Syntype	30
Paratype	9
Lectotype	6
Isoneotype	4
Neotype	4
Typus	3
Isosyntype	2
Isolectotype	1

**Table 6:** Main Chinese collectors before 1949.

Year	Collector	Collection Site
1918–1919	K.K.Tsoong = Guanguang Zhong	Baiyun Mountain
1919–1920	W.S. Kwok = Huaxiu Guo	Panyu
1929	C.L.Tso = Jinglie Zuo	Baiyun Mountain
1930	N.K.Chun = Nianqu Chen	Near Longan Cave
1929–1930	C.Wang = Zhi Wang	Baiyun Mountain, Longan Cave
1930	S.P.Ko = Xipeng Gao	Baiyun Mountain
1930	H.Y.Liang = Xiangri Liang	Baiyun Mountain
1927–1931	W.Y.Chun = Huanyong Chen	Near Longan Cave, Lingnan University (near Kangle Village, Haizhu District)
1930–1931	C.P.Lau = Zhuobin Liu	Near Dongshan, Sun Yat-sen University
1932, 1935	W.T.Tsang = Huaide Zeng	Conghua Sanjiao Mountain, Zengcheng
1928–1929, 1935–1937	Y.Tsiang = Ying Jiang	Conghua, Near Longan Cave, Baiyun Mountain
1937	S.Wang = Xiao Wang	Yuexiu
1937	S.K.Liou = Xinqi Liu	Panyu
1934, 1943–1944	F.C.How = Kuanzhao Hou	Baiyun Mountain

**Table 7:** Main collectors/collection teams after 1949.

Year	Collector/Collection Team	Collection Site
1948–1955, 1959–1960, 1962–1963, 1964–1988	Shaoqing Chen	Shipai, Baiyun Mountain, near Kangle village, Lvtian, and other places near Guangzhou
1950–1951	Yunzhang Gao	All over Guangzhou
1951–1957	Kuanzhao Hou	Around Guangzhou
1951	Hongda Zhang	All over Guangzhou
1951	Zhengyi Wu	All over Guangzhou
1951, 1953–1954	Baohan Liang	Guangzhou
1954–1956, 1958, 1963	Cheng Huang	Huadu, The old suburbs of Guangzhou (Baiyun Mountain)
1955–1958, 1963	Chengjiu Huang	Huadu
1956–1957	Yingguang Liu	The old suburbs of Guangzhou
1957, 1959	Wantao Lin	Conghua, Panyu
1958–1959	Sino-German collection team	Conghua
1958–1959, 1961–1964	Liang Deng	Conghua, The old suburbs of Guangzhou (South China Botanical Garden, Baiyun Mountain and other places)
1958	Zhi Huang	Conghua
1958	Zhaofen Wei	Zengcheng
1960–1961	Yourun Lin	Conghua
1960–1961	Mengzhen Li	Conghua
1961–1963	Ying Jiang	Around Guangzhou
1969–1970	Chinese herbal medicine group	The old suburbs of Guangzhou (Longan Cave, Huolu Mountain and other places)
1973	Xianrui Luo	Huadu
1979–1981, 2002, 2009	Huagu Ye	South China Botanical Garden, Conghua,
1981–1982	Fuwu Xing	South China Botanical Garden and nearby
1981–1982	Binghui Chen	South China Botanical Garden and nearby
2000–2008	Shijin Li	Huadu, Conghua, and other places
2001–2002	Yuehong Yan	Near Longan Cave
2002, 2004, 2005, 2009	Shixiao Luo	Guangzhou
2004	Bingqiang Xu	South China Botanical Garden
2005–2007	Shiyong Dong	All over Guangzhou
2009	Yushi Ye	All over Guangzhou

## Data Availability

The raw data that support the findings of this study are from public databases, such as the CVH (https://www.cvh.ac.cn/, accessed on 13 June 2023), the GBIF (https://www.gbif.org/, accessed on 1 July 2023), the Global Plants on JSTOR (https://plants.jstor.org/, accessed on 25 June 2023) and NSII (http://www.nsii.org.cn/2017/query.php?kingdom=plant, accessed on 9 June 2023).
